# Prevalence of positive screens for depression, anxiety, and PTSD symptoms among parents of infants admitted to a NICU: a pilot study

**DOI:** 10.3389/fpsyt.2026.1934224

**Published:** 2026-09-24

**Authors:** Adnan Ahmadiazad, Mohammed Saif Mohammed Nasir Ali, Hadeel Fathelrahman Ahmed Ali, Tahlil Abdalmahmoud Ibrahim, Ahmed Abdalla Abdalla, Mohamed Elsadig Elkhair, Anusha Sreejith

**Affiliations:** 1 College of Medicine, Gulf Medical University, Ajman, United Arab Emirates; 2 Department of Psychiatry, Al Amal Hospital, Dubai, United Arab Emirates; 3 Department of Community Medicine, College of Medicine, Gulf Medical University, Ajman, United Arab Emirates

**Keywords:** anxiety, depression, NICU (neonatal intensive care unit), parental health, PTSD - posttraumatic stress disorder

## Abstract

**Introduction:**

Parents of infants admitted to the Neonatal Intensive Care Unit (NICU) may experience substantial psychological distress. This study aimed to assess the prevalence of positive screens for depressive, anxiety, and post-traumatic stress disorder (PTSD) symptoms and to explore their associations with gender, education level, and parental age. The study also described the coping strategies reported by participants.

**Methods:**

This cross-sectional pilot study assessed 82 parents of infants admitted to the NICU at Thumbay University Hospital, Ajman. Parents who were primary caregivers and provided consent were included. Data were collected from March to July 2025 using self-administered questionnaires; Patient Health Questionnaire-9 (PHQ-9), Generalized Anxiety Disorder-7 (GAD-7), and PTSD Checklist for DSM-5 (PCL-5). They were then grouped based on whether they met the positive or negative screen criteria for the respective psychiatric symptoms.

**Results:**

Out of the 82 parents who had participated, 22% screened positive for anxiety symptoms, 13.4% for depression symptoms, and 9.8% for PTSD symptoms. Female participants had a higher proportion of positive screen for depressive symptoms than male participants (20.5% vs. 4.8% in men, p = 0.043), and younger participants (≤30 years) had a higher proportion of positive screens for PTSD symptoms than those aged >30 years (17.5% vs. 2.4%; Fisher’s exact p = 0.027), although the small number of positive cases warrants cautious interpretation. No significant associations were found with education level. The most reported coping strategies were religious/spiritual (34.8%) and physical/behavioral (26.1%) methods.

**Conclusion:**

A notable proportion of parents of infants admitted to the NICU in this UAE pilot cross-sectional study screened positive for symptoms of depression, anxiety, and PTSD, with female participants and younger participants showing higher proportions of positive screening for depressive and PTSD symptoms, respectively. These findings support consideration of mental health screening, promotion of a socially supportive environment, and culturally appropriate psychosocial support for parents of NICU-admitted infants.

## Introduction

Parents of infants admitted to the Neonatal Intensive Care Unit (NICU) may experience heightened psychological stress such as depression, anxiety, and post-traumatic stress disorder. Research has shown that complications like high-risk pregnancy, preterm birth, and other neonatal health issues predispose mothers to psychological distress, which can negatively affect their well-being, involvement in their infant’s care, and the well-being of the broader family. Such adverse effects may be mitigated through effective communication, education, and support for mothers, helping them adjust emotionally during their infant’s NICU admission (1).

Preterm delivery and NICU admission lead to significant emotional challenges for mothers. Stress levels are aggravated by factors such as separation from their infant, witnessing the baby’s illness, and the overwhelming NICU environment (2, 3). Persson C et al. identified that the most significant stressors for mothers include disruptions to their parental role, concerns over their infant’s condition, and the NICU environment itself. Stress levels were influenced by maternal age, duration of NICU stay, and infant-specific factors such as medical complications. Frequent maternal visits to the NICU were found to be associated with decreased levels of stress (2).

A study conducted in Northern Italy (4) compared mental health and participation of mothers and fathers in the care of NICU-admitted newborns. It showed that mothers reported higher levels of anxiety, depression, and stress compared to the fathers. 45.5% of mothers reported that they faced high stress compared to 24.1% of fathers, and 43.8% had depressed mood in contrast to 19% of the fathers. In addition, 76% of mothers had at least one mental health condition versus 45.3% of fathers. Mothers were observed to be more involved in caregiving activities. The study highlighted the importance of gender-specific support and mental health screening in order to achieve an improved parental well-being and a family-centered care. Similar findings were reported by Shetty et al. (5), with mothers of NICU-admitted infants reporting higher levels of anxiety (51%), depression (31%), and stress (41%) than fathers (26%, 12%, and 22%, respectively).

Higher levels of psychological distress have been reported among parents of preterm and low-birth-weight infants compared with parents of full-term infants. Mothers of preterm and low-birth-weight infants experienced higher levels of stress, anxiety, and depression than fathers, with contributing factors including delivery circumstances, maternal age, education level, and socioeconomic status (6, 7). The psychological impact on parents was not limited to the period of their infant’s hospitalization but was also reported after discharge, with studies similarly showing a higher mental health burden among mothers than fathers (8, 9). Additional factors, including parental age, infant gender, and length of NICU stay, were also associated with elevated levels of stress, anxiety, and depression and the persistence of these challenges after discharge (10, 11).

Post-traumatic stress symptoms have also been reported among parents of infants admitted to the NICU, particularly mothers. Traumatic childbirth, extended NICU stays, and infant complications were identified to be key factors associated with PTSD. Symptoms of PTSD were reported by both the mothers and fathers, with chronic effects. This thereby emphasizes the need for post-NICU psychological support and mental health screening (12–14).

Efforts to address the psychological impact on parents and families have led to the implementation of family-centered care and family-integrated care, where parents are actively involved in the care process with collaboration between families and healthcare providers in an attempt to create a more transparent and reliable communication channel (3). Research has also shown that having a supportive social environment served as a significant buffer against mental health complications in the setting of NICU (6, 7). Fathers have been shown to play an important role in supporting mothers, with greater paternal involvement associated with reduced risk of postpartum depression (15, 16). Multiple studies have highlighted the need for appropriate mental health support and early interventions to reduce the emotional burden on both parents (8, 9).

The psychological impact of NICU admission among patients and their caregivers is less understood in the Middle East region. This study aimed to assess the prevalence of positive screens for depressive, anxiety, and post-traumatic stress symptoms among parents of infants admitted to the NICU, examine their associations with selected demographic characteristics, and describe the coping strategies reported by participants.

## Methods

### Study design and setting

This cross-sectional study was conducted to assess parents of infants admitted to the Neonatal Intensive Care Unit (NICU) at Thumbay University Hospital, Ajman. The study population included adult parents of children who were currently admitted to the NICU and who provided informed consent. Parents who were not the primary caregivers of the NICU patient or who did not provide consent were excluded from the study. As this is a pilot study, a total of 82 parents were enrolled. The size of study was based on availability of eligible participants within the study duration.

### Data collection, instruments, and bias

Eligible parents of infants admitted to the NICU were recruited through convenience sampling between March and July 2025. Recruitment was limited to the predefined data-collection period, and eligible parents who were available during this period and consented to participate were enrolled. Members of the research team approached eligible parents within 96 hours of their infant’s admission to the NICU, explained the study objectives, and obtained written informed consent from those willing to participate. The questionnaires were then provided to participants and were completed and collected either immediately or within 24 hours. Both parents of the same infant could participate independently, as the screening instruments assessed each parent’s individual self-reported psychological symptoms. Participants completed a questionnaire in English consisting of demographic questions, the PHQ-9, GAD-7, and PCL-5. Completed questionnaires were collected by the research team for analysis. For participants who preferred Arabic, a native Arabic-speaking medical doctor proficient in English verbally translated the questionnaire items and response options during administration and recorded the participant’s responses on the corresponding standardized English-language forms. The language of administration was not separately recorded.

To promote honest reporting and reduce response bias, questionnaires were completed anonymously, and no personal identifying information was collected. Participants were provided privacy and adequate time to complete the questionnaires at their convenience. Participation was entirely voluntary, and participants retained the right to decline or discontinue participation at any stage without consequence. Standardized screening instruments included the Patient Health Questionnaire-9 (PHQ-9) and Generalized Anxiety Disorder-7 (GAD-7), administered using the combined English UHS Rev 4/2020 form (17). The English PHQ-9 and GAD-7 have demonstrated established reliability and validity in previous psychometric evaluations (18). The PTSD Checklist for DSM-5 (PCL-5; Standard Form, version date 29 August 2023) was used in the study as well (19). The PCL-5 has demonstrated established reliability and validity in previous psychometric evaluation (20, 21).

No formal sample-size or power calculation was performed, and the study was not specifically powered to detect associations between demographic characteristics and psychological screening outcomes. The study size was determined by the number of eligible participants available during the study period and was intended as an exploratory pilot study. For exploratory subgroup analysis, parental age was dichotomized at 30 years. This cut-off was selected during data analysis because it was close to the central age distribution of the collected sample (mean 31.98 years, Standard Deviation (SD) 5.99; median 31 years) and produced approximately balanced groups (≤30 years, n=40; >30 years, n=42). The 30-year threshold does not represent an established clinical cut-off.

Coping strategies were assessed using an open-ended question asking participants how they coped with the emotional challenges related to their infant’s NICU admission. No predefined response options or coping categories were provided to participants. The responses were reviewed and grouped *post hoc* into five descriptive categories based on conceptual similarity: religious/spiritual coping, physical/behavioral coping, avoidant/passive coping, professional/social support, and work/distraction coping. These categories were developed pragmatically to group similar responses. They were not derived from a validated coping scale or classification framework. Coping strategies were analyzed descriptively, and no inferential analyses were performed between coping categories and psychological screening outcomes.

### Statistical analysis

Statistical analysis was performed using SPSS version 29. Categorical variables were summarized using frequencies and percentages. Participants were classified as screening positive or negative according to established cut-off values for PHQ-9, GAD-7, and PCL-5. Associations between demographic variables (gender, age group, education level) and screening outcomes were assessed using the Chi-square test when assumptions were met. Associations between categorical variables were assessed using Fisher’s exact test when more than 20% of expected cell frequencies were <5 or when any expected cell frequency was <1. All tests were two-sided. Unadjusted odds ratios (ORs) with corresponding 95% confidence intervals (CIs) were calculated directly from the contingency tables to quantify the strength of associations. No multivariable adjustment was performed. A p-value < 0.05 was considered statistically significant. Missing responses were excluded from the relevant analyses, and no imputation was performed. Because not all participants completed every demographic or coping item, analyses were conducted using available valid responses for each variable. Participants with missing responses for a specific demographic or coping variable were excluded only from the relevant analysis. Therefore, denominators vary slightly across demographic and subgroup analyses.

The following screening criteria were used based on cut-offs that are widely validated and have been applied to our analysis. It is crucial to note that we used the screening criteria only to assess the risk of positive screen for symptoms of depression, anxiety, and PTSD and not to confirm a diagnosis.

•⁠ PCL-5 ≥ 33 → positive screen for PTSD symptoms (21, 22).
•⁠ PHQ-9 ≥ 10 → positive screen for depression symptoms (23, 24).
•⁠ ⁠GAD-7 ≥ 10 → positive screen for anxiety symptoms (25, 26).

### Ethics

Ethical approval for this study was obtained from the Institutional Review Board of Gulf Medical University (Ref. no. IRB-COM-FAC-112-Mar-2025).

## Results

The total pilot-study sample was 82. Percentages were calculated based on valid responses for each variable. Gender data were missing for 1 participant, and education data were missing for 9 participants. Gender data were available for 81 participants, age data for 82 participants, and education data for 73 participants. Among participants with available gender data, 51.9% were male and 48.1% were female. For age, 51.2% were older than 30 years, while 48.8% were 30 years or younger. The mean participant age was 31.98 years (SD 5.99), with a median age of 31 years (range 20–45 years). Among participants who provided education data, 79.5% held a bachelor’s degree or higher, while 20.5% had completed high school education (**Table 1**).

**Table 1:** Demographic characteristics of participants.

Variable	Category	Frequency (N)/Value	Percentage (%)
Gender	Male	42	51.9
	Female	39	48.1
Age (years)	Mean ± SD	31.98 ± 5.99	-
	Median (range)	31 (20–45)	-
Age Group	≤ 30 years	40	48.8
	> 30 years	42	51.2
Education Level	High School	15	20.5
	Bachelor’s and above	58	79.5

Percentages are based on valid responses for each screening instrument. Valid responses were available for 82 participants for GAD-7, PHQ-9, and PCL-5. Positive screening thresholds were defined as GAD-7 ≥10, PHQ-9 ≥10, and PCL-5 ≥33. For anxiety symptoms (GAD-7), 64 participants (78%) screened negative and 18 (22%) screened positive. For depression symptoms (PHQ-9), 71 participants (86.6%) screened negative and 11 (13.4%) screened positive. For PTSD symptoms (PCL-5), 74 participants (90.2%) screened negative and 8 (9.8%) screened positive. 95% confidence intervals (CIs) for prevalence estimates were calculated using the Wilson score method (**Table 2**).

**Table 2:** Overall prevalence of positive screens.

Measure	Negative N (%)	Positive N (%)	95% CI
Anxiety (GAD-7)	64 (78.0%)	18 (22.0%)	14.4–32.1%
Depression (PHQ-9)	71 (86.6%)	11 (13.4%)	7.7–22.4%
PTSD (PCL-5)	74 (90.2%)	8 (9.8%)	5.0–18.1%

Percentages are based on participants with available gender data and valid screening scores for each measure. Participants with missing gender data were excluded from this subgroup analysis. Odds ratios (ORs) are reported with 95% confidence intervals (CIs), with females as the reference group. p-values were calculated using the Chi-square test when appropriate, and Fisher’s exact test when appropriate. Statistical significance = p < 0.05. A statistically significant association was observed between gender and positive screen for depressive symptoms (OR = 0.194, 95% CI 0.038–0.978; Fisher’s exact p = 0.043), with female participants reporting a higher proportion of positive PHQ-9 screens. Anxiety and PTSD positive screen proportions were descriptively higher among female participants, but these associations were not statistically significant (**Table 3**).

**Table 3:** Gender and screening outcomes (PHQ-9, GAD-7, PCL-5).

Measure	Gender	Negative N (%)	Positive N (%)	OR (male vs female)	95% CI	p-Value
Depression (PHQ-9)	Male	40 (95.2)	2 (4.8)	0.194	0.038–0.978	0.043
	Female	31 (79.5)	8 (20.5)			
Anxiety (GAD-7)	Male	36 (85.7)	6 (14.3)	0.424	0.140–1.289	0.124
	Female	28 (71.8)	11 (28.2)			
PTSD (PCL-5)	Male	38 (90.5)	4 (9.5)	0.921	0.21–3.97	1.000
	Female	35 (89.7)	4 (10.3)			

Age data were available for all 82 participants. p-values were calculated using the Chi-square test when assumptions were met, and Fisher’s exact test when appropriate. Statistical significance = p < 0.05. Odds ratios (ORs) with 95% confidence intervals (CIs) were calculated to estimate the precision of associations between age group and positive screening outcomes, with participants aged >30 years as the reference group. An exploratory association was observed between younger age (≤30 years) and positive screen for PTSD symptoms (OR = 8.70, 95% CI 1.02–74.4; Fisher’s exact p = 0.027). No statistically significant associations were observed for depression or anxiety screening outcomes, although positive-screen proportions were descriptively higher among younger participants (**Table 4**).

**Table 4:** Age group and screening outcomes.

Measure	Age group	Negative N (%)	Positive N (%)	OR (≤30 vs >30 years)	95% CI	P-value
Anxiety (GAD-7)	≤ 30 yrs	31 (77.5)	9 (22.5)	1.06	0.37–3.03	0.907
	> 30 yrs	33 (78.6)	9 (21.4)			
Depression (PHQ-9)	≤ 30 yrs	33 (82.5)	7 (17.5)	2.02	0.54–7.49	0.289
	> 30 yrs	38 (90.5)	4 (9.5)			
PTSD (PCL-5)	≤ 30 yrs	33 (82.5)	7 (17.5)	8.70	1.02–74.4	0.027
	> 30 yrs	41 (97.6)	1 (2.4)			

Percentages are based on participants with available education data and valid screening scores for each measure. Participants with missing or unclassified education data were excluded from this subgroup analysis. Odds ratios (ORs) with 95% confidence intervals (CIs) were calculated using participants with a bachelor’s degree or above as the reference group. p-values were calculated using the Fisher’s exact test. Statistical significance = p < 0.05. Education level was not significantly associated with positive screening for depressive, anxiety, or PTSD symptoms. Positive-screen proportions for depressive and anxiety symptoms were descriptively higher among participants with a bachelor’s degree or above, while positive-screen proportions for PTSD symptoms were slightly higher among those with high-school education (**Table 5**).

**Table 5:** Education and screening outcomes.

Measure	Education level	Negative N (%)	Positive N (%)	OR (high school vs bachelor’s and above)	95% CI	P-value
Depression (PHQ-9)	High School	14 (93.3)	1 (6.7)	0.45	0.05–3.88	0.674
	Bachelor’s and above	50 (86.2)	8 (13.8)			
Anxiety (GAD-7)	High School	13 (86.7)	2 (13.3)	0.53	0.11–2.67	0.720
	Bachelor’s and above	45 (77.6)	13 (22.4)			
PTSD (PCL-5)	High School	13 (86.7)	2 (13.3)	1.63	0.28–9.37	0.627
	Bachelor’s and above	53 (91.4)	5 (8.6)			

Coping data were available for 69 of 82 participants (84.1%). Thirteen participants did not provide a coping response and were excluded from this analysis.

Religious/spiritual coping (34.8%) and physical/behavioral coping (26.1%) were most frequently reported. Avoidant/passive coping represented 18.8% of valid responses. No inferential analyses were conducted between coping style and screening outcomes (**Table 6**).

**Table 6:** Coping mechanisms reported by participants.

Coping category	Examples	N (%)
Religious/Spiritual Coping	Prayers, meditation, reading Quran	24 (34.8)
Physical/Behavioral Coping	Exercise, sleep, sports, relaxation	18 (26.1)
Avoidant/Passive Coping (incl. “none”, “no stress”)	Nothing, avoidance, smoking	13 (18.8)
Professional/Social Support	Professional help, communication, family support	9 (13.0)
Work/Distraction Coping	Work, reading, shopping	5 (7.2)
Total		69 (100.0)

## Discussion

### Overview of the study

Our study is one of the first cross-sectional studies from the United Arab Emirates to examine symptoms of anxiety, depression, and post-traumatic stress among parents of infants admitted to a neonatal intensive care unit (NICU) in Thumbay University Hospital, Ajman.

In our sample of 82 participants with nearly equal gender distribution using valid screening tools, we found that 22.0% screened positive for anxiety symptoms, 13.4% for depressive symptoms and 9.8% for PTSD symptoms. Female participants had a higher proportion of positive screens for depressive symptoms (p = 0.043), and younger participants (≤30 years) had a higher proportion of positive screens for PTSD symptoms (p = 0.027). Education level was not significantly associated with positive screening for depressive, anxiety, or PTSD symptoms, although participants with higher education tended to have slightly higher depressive and anxiety symptoms, while PTSD symptoms were slightly higher among those with lower education. Religious or spiritual coping was the most commonly reported coping strategy (34.8%) among parents, followed by physical/behavioral coping like exercise (26.1%).

### Comparison with international literature

In comparison with recent international systematic reviews and meta-analyses, our findings were consistent in showing that parents of NICU infants experience psychological distress, including anxiety, depression, and trauma-related symptoms. In contrast, the prevalence rates of positive screens in our study were lower than those reported in pooled analyses, which found anxiety symptoms in approximately 42% of parents during the early weeks of their infant’s admission (27) and around 51% of mothers and 26% of fathers in combined estimates (5). In our sample, 22% of parents screened positive for anxiety symptoms. This difference may be partly explained by the timing of assessment as our cross-sectional design captured a single point, whereas several previous studies assessed parents during their first days of admission when anxiety tends to peak, or as it decreases with time close to discharge. Other possible explanations include the use of brief screening tools rather than structured diagnostic interviews that capture more symptoms; cultural differences in emotional expression and fear of stigma acting as a barrier to disclosure; as well as individual differences in infant morbidity and other possible bio-psycho-social factors.

### Depression and sex differences

Positive screening for depressive symptoms was observed in 13.4% of participants. This rate is comparable to ranges reported in prior international studies. In this exploratory analysis, female participants had a higher proportion of positive screens for depressive symptoms than male participants, consistent with prior literature identifying higher emotional vulnerability and caregiving burden among mothers of NICU infants (5). This association was unadjusted and should not be interpreted as evidence of an independent effect of sex. Potential explanations include the peripartum biological and psychosocial burden on mothers, greater caregiving responsibilities, and culturally identified roles that increase emotional load and worry (5). Evidence from recent meta-analytic work also confirms that depression is more prevalent among mothers of NICU infants, with pooled estimates showing higher depression severity in mothers (ES = 0.31) compared to fathers (ES = 0.12) (5). Similarly, large population-based data from England report that nearly one in four mothers whose infants required neonatal care experienced postnatal depression at six months (28).

### Parental age and PTSD symptoms

An exploratory association was observed between younger age and positive PTSD screening in our sample. However, this finding should be interpreted cautiously because only eight participants screened positive for PTSD symptoms, including only one participant aged >30 years. However, evidence supporting “younger age” as a risk factor in NICU parents is limited. A recent systematic review identified other key risk factors for parental PTSD including being female, perceiving one’s infant as severely ill, previous obstetric complications, existing mental-health diagnoses, and insufficient psychological support in the NICU (29). Given the small number of positive cases and the absence of adjustment for potential confounders, the observed age association should not be interpreted as evidence that younger age is an independent risk factor. Further adequately powered studies are needed to clarify whether parental age is associated with PTSD symptoms in this population.

### Coping strategies

Religious and spiritual coping was the most frequently reported strategy in our cohort (34.8%), followed by physical and behavioral coping (26.1%). This finding aligns with the broader cultural and religious context in the region, where spirituality often plays a central role in how families cope in difficult times. Previous literature suggests that experiences such as a child’s illness or hospitalization are often seen through the lens of divine will and viewed as a test of patience and faith, which may support acceptance and resilience. A recent study of mothers in a neonatal intensive care unit in Türkiye found a significant positive correlation between positive religious coping and parental beliefs (β = 0.256, p = 0.005) (30). However, our study did not examine associations between coping strategies and psychological screening outcomes. In practice, this highlights the importance of culturally attuned psychosocial support for parents in NICUs, where understanding and respecting families’ spiritual frameworks may strengthen trust and enhance engagement with care.

### Education level and psychological outcomes

No statistically significant associations between education level and screening outcomes were detected in this sample. Given the limited statistical power, these findings should not be interpreted as evidence that no association exists. Although education level was not significantly associated with any of our screening outcomes: positive-screen proportions for depressive and anxiety symptoms were descriptively higher among participants with a bachelor’s degree or above, whereas the proportion screening positive for PTSD symptoms was slightly higher among those with high-school education. Recent studies have found that education and tolerance for uncertainty are important for parental and psychological wellbeing while suggesting that higher education may offer more cognitive and resource advantages under stressful circumstances (31, 32); however, tolerance for uncertainty and these potential explanatory mechanisms were not assessed in our study.

### Strengths and limitations

Key strengths of our study include the use of validated screening tools, inclusion of both male and female participants, and the collection of a brief but informative data on coping strategies.

Our study has a few limitations: being cross-sectional, we had limited ability to assess causality or changes in stress over time; we relied on self-report screening tools rather than structured clinical interviews; parents were understandably in distress, which made it challenging for them to complete longer assessments; our sample size is modest and drawn from a single hospital, which limits generalizability to other hospitals and the broader population. The use of convenience sampling may also have introduced selection bias, as participants who were available and willing to participate during the predefined study period may not be representative of all parents of NICU-admitted infants in the UAE. Some parents did not complete all questionnaire parts, with coping data missing for 13 of the 82 participants (16%) which may have influenced the observed coping strategies. Although outcomes were individually self-reported, parents of the same infant may share common NICU-related experiences; therefore, potential within-family correlation cannot be excluded. The modest sample size and small number of positive screening outcomes limited statistical power for subgroup analyses; therefore, non-significant findings should not be interpreted as evidence of no association.

In addition, infant- and NICU-related clinical characteristics, including gestational age, birth weight, prematurity status, severity of illness, and length of NICU stay, were not recorded. These factors may influence parental psychological symptoms and therefore represent potential unmeasured confounders that could not be examined in our study. Additionally, the subgroup associations were unadjusted, and no multivariable analysis was performed; therefore, potential confounding cannot be excluded.

A proportion of parents in our UAE sample screened positive for anxiety, depression, and PTSD symptoms, with a higher proportion of positive depressive-symptom screens among female participants and positive PTSD-symptom screens among younger participants. These findings support the consideration of screening, psychoeducation, trauma-informed care, routine identification, and culturally appropriate support for parents in the NICU. Larger studies are needed to evaluate the potential benefits of structured screening and support pathways for parents and families.

## Conclusion

This study identified a notable proportion of parents of NICU-admitted infants who screened positive for depressive, anxiety, and PTSD symptoms in the UAE. We identified associations between female gender and positive screening for depressive symptoms and between younger age (≤30 years) and positive screening for PTSD symptoms; however, these findings should be interpreted cautiously given the modest sample size. Despite our study being limited by its descriptive design and small sample size, it fills an important gap in the Middle East data and supports consideration of mental health screening and appropriate psychosocial support pathways for parents of NICU-admitted infants.

Additionally, it highlights the relevance of cultural and religious understanding when engaging with the parents. Larger, multicenter studies would be warranted to further confirm these findings and conduct advanced statistical comparisons, including multivariable analyses.

## Data Availability

The raw data supporting the conclusions of this article will be made available by the authors, without undue reservation.
